# Proscillaridin A exerts anti-tumor effects through GSK3β activation and alteration of microtubule dynamics in glioblastoma

**DOI:** 10.1038/s41419-018-1018-7

**Published:** 2018-09-24

**Authors:** Raphael Berges, Emilie Denicolai, Aurélie Tchoghandjian, Nathalie Baeza-Kallee, Stephane Honore, Dominique Figarella-Branger, Diane Braguer

**Affiliations:** Aix-Marseille Univ, CNRS, INP, Inst Neurophysiopathol, Marseille, France

## Abstract

Glioblastoma (GBM) is characterized by highly aggressive growth and invasive behavior. Due to the highly lethal nature of GBM, new therapies are urgently needed and repositioning of existing drugs is a promising approach. We have previously shown the activity of Proscillaridin A (ProA), a cardiac glycoside inhibitor of the Na(+)/K(+) ATPase (NKA) pump, against proliferation and migration of GBM cell lines. ProA inhibited tumor growth in vivo and increased mice survival after orthotopic grafting of GBM cells. This study aims to decipher the mechanism of action of ProA in GBM tumor and stem-like cells. ProA displayed cytotoxic activity on tumor and stem-like cells grown in 2D and 3D culture, but not on healthy cells as astrocytes or oligodendrocytes. Even at sub-cytotoxic concentration, ProA impaired cell migration and disturbed EB1 accumulation at microtubule (MT) plus-ends and MT dynamics instability. ProA activates GSK3β downstream of NKA inhibition, leading to EB1 phosphorylation on S155 and T166, EB1 comet length shortening and MT dynamics alteration, and finally inhibition of cell migration and cytotoxicity. Similar results were observed with digoxin. Therefore, we disclosed here a novel pathway by which ProA and digoxin modulate MT-governed functions in GBM tumor and stem-like cells. Altogether, our results support ProA and digoxin as potent candidates for drug repositioning in GBM.

## Introduction

Cardiac glycosides (CG) are a large family of natural compounds that are well-known drugs for increasing cardiac contractile force in cardiac diseases. Proscillaridin A (ProA) is a familiar drug that belongs to the bufadienolide chemical sub-group. In cardiomyocytes, CG bind and inhibit the sodium (Na^+^)/potassium (K^+^)-ATPase (NKA) transmembrane pump. The consecutive elevation of the intracellular Na^+^ level stimulates the Na^+^/Ca^2+^ exchanger mechanism. As a result, the intracellular Ca^2+^ concentration is increased, promoting cellular events such as myocardial contractibility, leading to the positive inotropic effects of the CG^1^. The anticancer effects of CG were suggested in 1979 by Stenkvist in a study of women treated with *digitalis* in combination with chemotherapy for breast cancer^2^. A higher survival rate was also observed in a long-term follow-up study^3^. Thereafter, anticancer effects of different CG were shown on several cell lines and in various in vivo models^4^. However, sensitivity of CG on cell proliferation and viability depend on tumor type and CG may not be good candidates for cancer therapeutics in all tumors^5^. Hence, the mechanism of the anti-cancer activity of CG needs to be deciphered. The ability of CG to inhibit NKA pump function resulting in increased Ca^2+^ concentration and subsequent apoptosis was first suggested^6^. Furthermore, activation of NKA as a signal transducer in cell signaling pathways has been proposed to explain the anticancer activity of CG at low nanomolar concentrations, which do not lead to calcium overload^7^. More recently, additional intracellular targets for CG, whose modulation might be off-NKA targeting, have been described such as inhibition of transcription factor activity and immunogenic cell death induction^4^.

In our previous study, ProA was the best candidate molecule selected by high throughput screening for anticancer activity against glioblastoma (GBM) cell lines^8^. The Prestwick chemical library® was screened for anti-proliferative and anti-migratory properties towards two human primary GBM stem-like cell lines, GBM6 and GBM9, previously established and characterized in our laboratory^9^. These cancer stem-like cell lines represent two appropriate study models of GBM (i.e., mesenchymal and proneural, respectively)^10^. ProA showed cytotoxic properties, induced G2/M phase blockage, triggered cell death by apoptosis, and impaired GBM self-renewal capacity even at low concentrations. Moreover, ProA controlled tumor growth in vivo and increased mice survival after orthotopic transplantation of U87-MG and GBM6 cells^8^. Interestingly, preliminary personal data indicate that ProA affected microtubule (MT) network in GBM cell lines in a concentration-dependent manner.

MTs are major cytoskeletal component which exhibit a crucial dynamic process. Indeed, MT plus-ends undergo continuous cycles of polymerization (growth) and depolymerization (shrinkage), with periods of pauses, a process referred to as “dynamic instability”^11,12^. The transition between MT growth and shrinkage is defined as catastrophe, and a rescue defines the switch from shortening to growth. Growing MT plus-ends serve as transient binding platforms for essential proteins that regulate MT dynamics and their interactions with cellular substructures during migration and segregation of chromosomes towards cell poles during mitosis^13^. Among these proteins, the end-binding protein EB1 is a MT-plus-end-tracking protein (+TIP) that has the intrinsic ability to bind only to the tips of growing MT ends to recruit networks of interacting partners. During MT polymerization, new high affinity binding sites for EB1 are generated at MT plus-ends. These high affinity binding sites exist for a period of time and then progressively disappear from the MT lattice, making the binding of EB1 resembling to a comet.

MT dynamics are the target of a Microtubule-Targeting Agents (MTAs) which display a dose-dependent anti-proliferative effect. At high concentrations, MTAs are cytotoxic; they inhibit cell proliferation by suppressing dynamicity of spindle MTs, which are essential for proper chromosome separation during cell division, subsequently inducing a mitotic blockage and finally cell death by apoptosis^11^. At sub-cytotoxic concentrations, MTAs exert anti-migratory activity in several tumor cell lines, including GBM cells, GBM6 stem-like cells, and endothelial cells^14–16^. The anti-migratory effect was associated with a reduced accumulation of EB1 and other +TIPs at MT ends and an increase in distance-based MT catastrophe frequencies that led to the reduction of the number of MTs targeting to adhesion sites, thus inhibiting cell migration^14,17^.

All these data prompted us to investigate whether the mechanism of action of ProA in GBM is MT-dependent. ProA was cytotoxic for GBM cell lines and cancer stem-like cells (GBM6) but not for healthy brain cells. At sub-cytotoxic concentrations, ProA induced an alteration of MT dynamic instability. It resulted from GSK3β activation following binding of ProA on NKA, leading to EB1 phosphorylation and subsequent reduction of EB1 accumulation at MT plus-end, and finally inhibition of cell migration. Taken together, our results show that ProA mimics the anticancer activity of MTA in GBM.

## Results

### ProA displays cytotoxic and anti-migratory properties on GBM cell lines including cancer stem like-cells, but not on healthy neural cells

**Fig. 1:**
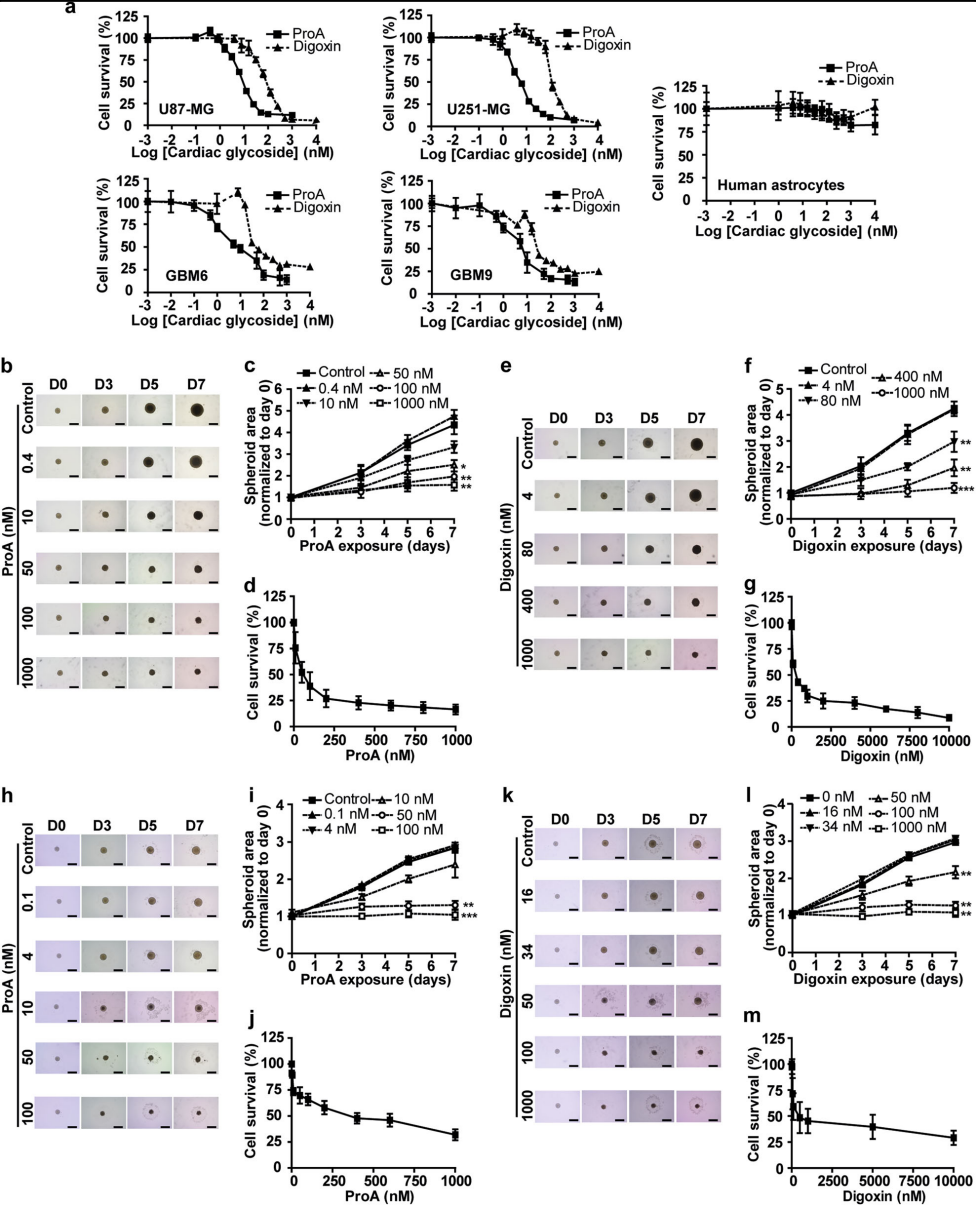
ProA displayed cytotoxic properties on GBM cell lines, but not on healthy neural cells. Dose-response curves of the cytotoxicity of ProA and digoxin in U87-MG, U251-MG, GBM6, GBM9 and human astrocytes (**a**). At least three independent experiments were performed. Three-dimensional (3D) tumor spheroids of U87-MG (**b**–**g**) and GBM6 cells (**h**–**m**) were treated with indicated concentrations of ProA (**b**–**d** and **h**–**j**) and digoxin (**e**–**g** and **k**–**m**) for 7 days. Bright-field photomicrographs (**b**, **e**, **h**, **k**), dose-dependance and time-dependance on spheroid area (**c**, **f**, **i**, **l**) and viability at day 7 of exposure, assessed by resazurin reduction test (**d**, **g**, **j**, **m**). Bar = 500 μm. At least three independent experiments were performed. Bar ± SEM. *p < 0.05; **p < 0.005; ***p < 0.001

A dose-response cytotoxicity assay of ProA (0–10^4^ nM) was conducted for 72 h on GBM cell lines (U87-MG and U251-MG), cancer stem-like cells (GBM6 and GBM9), and healthy brain cells (astrocytes and oligodendrocytes). Digoxin, which belongs to the cardenolide subgroup of CG, was tested for comparison (Fig. 1a, Supplementary Fig.1). The drug concentrations required to reduce viability by 50% (EC50) and 100% (EC100), together with the higher non-cytotoxic concentration (EC0) were determined after 72 h treatment (Table 1). Both ProA and digoxin were found to be toxic at nanomolar concentrations and ProA was around 10-fold more potent than digoxin. Furthermore, bufalin and digitoxin, bufadienolide and cardenolide compounds respectively, were found to be cytotoxic at low concentrations (Table 1). Importantly, no cytotoxicity was detectable on astrocytes or oligodendrocytes in a large range of concentrations of drugs. These data suggest a specific sensitivity of GBM tumor cells towards CG, without side effects on neural healthy cells from human, mouse or rat origin.

**Table 1:** EC0, EC50, and EC100 values of ProA, digoxin, bufalin, and digitoxin in U87-MG, U251-MG, GBM6, and GBM9 cell lines determined by MTT assay at 72 h treatment. EC0: sub-cytotoxic concentration, EC50 and EC100: concentrations reducing by 50 and 100% cell survival, respectively

	ProA			Digoxin			Bufalin	Digitoxin
	EC0 (nM)	EC50 (nM)	EC100 (nM)	EC0 (nM)	EC50 (nM)	EC100 (nM)	EC50 (nM)	EC50 (nM)
U87-MG	0.4	9.7 ± 1.3	100	4	79.9 ± 6.1	1000	2.4 ± 1.0	30.9 ± 1.0
U251-MG	0.4	4.7 ± 1.8	100	16	124.6 ± 0.8	1000	–	–
GBM6	0.1	4.0 ± 1.2	100	16	33.8 ± 1.6	1000	3.1 ± 1.2	41.4 ± 1.1
GBM9	0.1	4.0 ± 1.4	100	9	20.7 ± 1.4	1000	–	–

Similar results were obtained on 3D cell cultures in spheroid form, which are more representative of tumors as their cell morphology and phenotype are similar to those found in the original tissue architecture. Three-dimensional (3D) tumor spheroids derived from U87-MG (Fig. 1b–g) were treated with different concentrations of ProA (Fig. 1b–d) and digoxin (Fig.1e–g) up to 7 days. Concentrations of ProA at 50 nM and above exhibited smaller cross-sectional areas as compared to controls (Fig.1b, c). Indeed, after 7 days of treatment, 50, 100 and 1000 nM ProA significantly reduced spheroid size by 42.4 ± 5.1, 54.8 ± 5.7, and 63.7 ± 3.6% compared with control (Fig. 1b, c). In the same manner, 80, 400, and 1000 nM digoxin reduced spheroid size by 30.1 ± 9.2, 53.8 ± 7.6, and 71.9 ± 4.5% compared with control (Fig. 1e, f). Moreover ProA and digoxin decreased cell viability in a dose-dependent manner (Fig. 1d, g). The same dose-effects and time-effects on spheroid areas and viability were obtained using spheroids produced from GBM6 cells (Fig. 1h–m). Together, these data highlight the potent cytotoxic effect of ProA and digoxin on GBM.

**Fig. 2:**
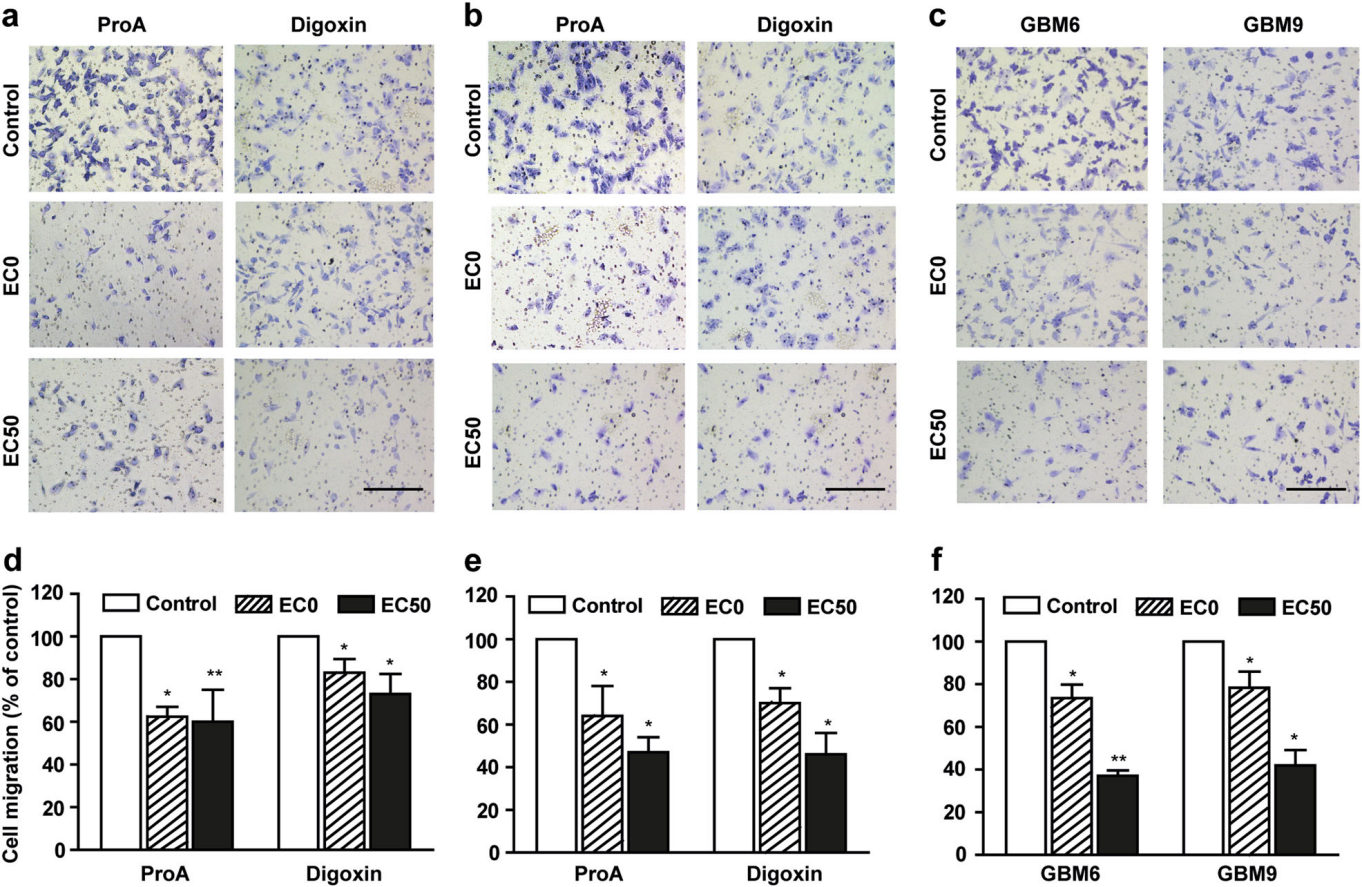
ProA displayed antimigratory properties on GBM cell lines. Representative images and quantification of U87-MG (**a**, **d**), U251-MG (**b**, **e**) migratory cells treated with vehicle (control), ProA and digoxin (at EC0 and EC50) for 5 h using the transwell migration assay. Representative images and quantification of migratory cancer stem-like cells GBM6 and GBM9 (**c**, **f**) treated with vehicle (control) or ProA (at EC0 and EC50) for 5 h using the transwell migration assay. Bar = 200 μm. Quantification was expressed as percentage of migrating cells relative to 100% of control cells. At least three independent experiments were performed for each condition. Bar ± SEM. *p < 0.05; **p < 0.005

Inhibition of cell migration by ProA and digoxin was assessed by using a transwell assay (Fig. 2). A dose-effect was shown, that was statistically significant even at low and sub-cytotoxic concentration (EC0). Indeed, 36.7 ± 4.7%, 36.0 ± 14.0%, 28.7 ± 4.8%, and 21.6 ± 7.5% of respectively U87-MG, U251-MG, GBM6, and GBM9 cell migration was inhibited at EC0. Similar effects were observed using digoxin at equipotent concentrations.

### ProA inhibits EB1 accumulation at MT plus-ends and increased distance-based catastrophe frequency

**Fig. 3:**
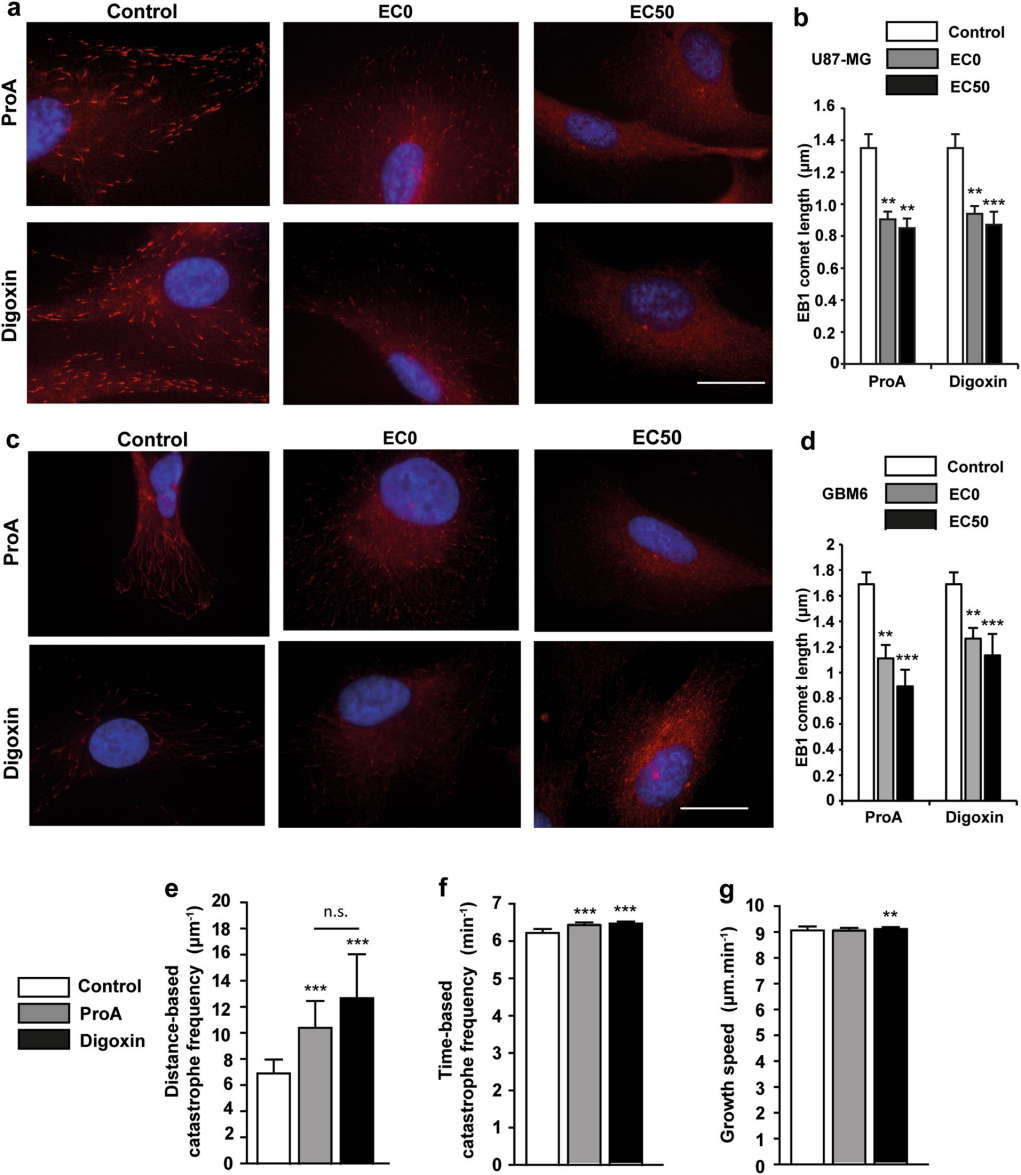
ProA inhibited EB1 accumulation at MT plus-ends and increased distance-based catastrophe frequency. Immunofluorescence staining of EB1 (red) in U87-MG (**a**) and GBM6 (**c**) treated or not (control) with ProA and digoxin at EC0 and EC50 for 5 h. Bar = 10 μm. Quantification of EB1 comet length in U87-MG (**b**) and GBM6 (**d**) treated with vehicle (control), ProA and digoxin at EC0 and EC50 for 5 h. Measurement of MT dynamics parameters by live microscopy of EB3 at MT plus-ends in EB3-GFP transfected U87-MG cells exposed to vehicle (control), ProA and digoxin at EC50 for 5 h. EB1 comets were tracked over time to measure MT distance-based and time-based catastrophe frequency (**e**–**g**). Bar ± SEM. *p < 0.05; **p < 0.005; ***p < 0.001; n.s., *p* > 0.05

Preliminary experiments showed that ProA and digoxin altered global MT cytoskeleton architecture by immunofluorescence staining of tubulin. At EC50, U87-MG or GBM6 cells lost their pronounced MT network which became disrupted and disorganized, in comparison with control untreated cells. Same results were observed with bufalin and digitoxin on U87-MG and GBM6 cells (Supplementary Fig. 2). Otherwise, MTAs are known to affect cell viability and migration via inhibition of EB1 accumulation at MT plus-ends and alteration of MT dynamics instability at low concentrations^14,16,17^. Here, we intended to understand whether ProA and digoxin lead to such processes. Then, we examined the intracellular localization of EB1, a +TIP that regulates MT dynamic instability. Immunofluorescence microscopy revealed a typical pattern of EB1 with comet-like structures at the plus-ends of MTs in U87-MG and GBM6 control cells (Fig. 3a, c). Treatment with ProA at the sub-cytotoxic concentration (EC0) for 5 h significantly decreased the EB1 comet length from 1.35 ± 0.09 µm in U87-MG control cells to 0.90 ± 0.05 (*p* < 0.005) after treatment, and from 1.67 ± 0.11 µm in GBM6 control cells to 1.16 ± 0.13 (*p* < 0.005) after treatment (Fig. 3b, d). Digoxin at equipotent concentration also decreased EB1 comet length in both cell lines. To evaluate the effect of CG on EB1 comet length and MT dynamics in cells, we used live imaging confocal microscopy to track all MT plus-ends positions in the cytoplasm of U87-MG cells transfected with EB3-GFP after ProA and digoxin treatment (Fig. 3e–g). A strong increase was reported in the distance-based catastrophe frequency (+50.1% and +83.5% for ProA and digoxin respectively, *p* < 0.001). Slight increase in the time-based catastrophe frequency was also observed which was statistically significant due to the high number of analyzed tracks (more than 1 × 10^4^ per condition). This slight effect on the time-based catastrophe frequency is explained by a very poor or no effect on MT growth speed. These results indicate that ProA and digoxin, like MTAs, are able to alter EB comet length at growing ends of MTs, thus increasing MT catastrophes in GBM cells.

### ProA does not inhibit pure tubulin polymerization

**Fig. 4:**
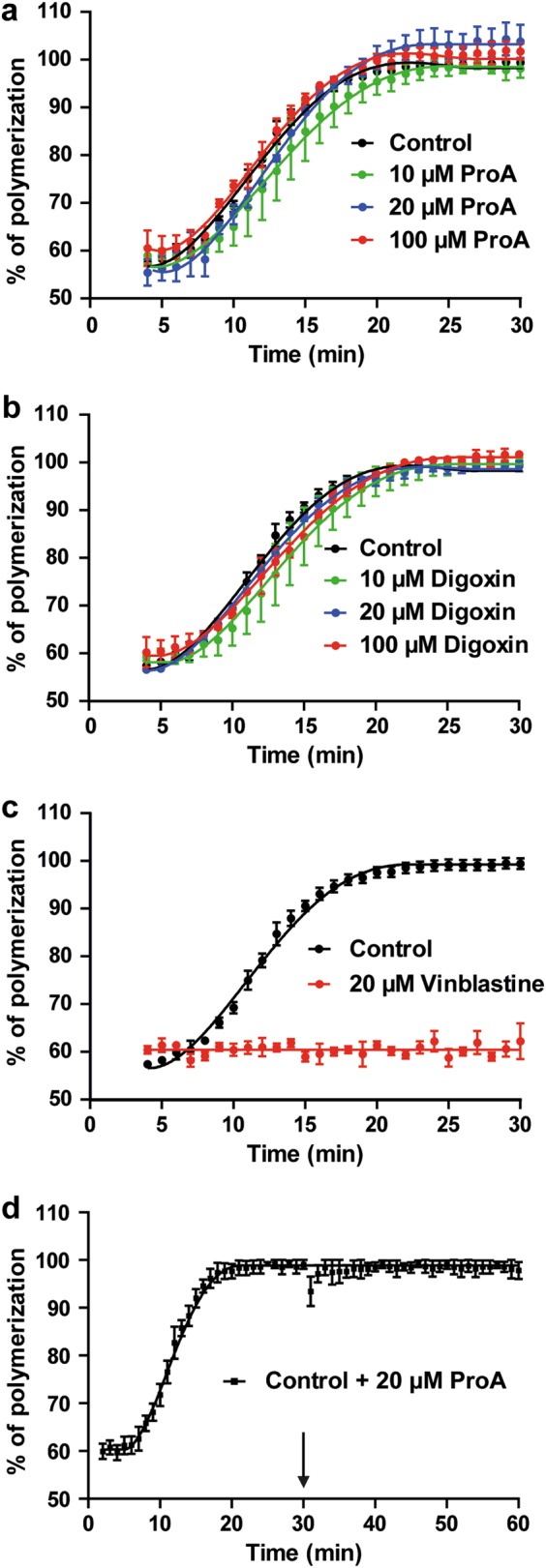
ProA did not inhibit pure tubulin polymerization. ProA (**a**), digoxin (**b**) or vehicle (control) was added at different concentrations (10, 20, and 100 µM) to unpolymerized tubulin, and the percentage of MT assembly was quantified by turbidimetry after 30 min. Vinblastine (20 µM) was used as positive control (**c**). When ProA (20 µM) was added to fully polymerized MTs (arrow), no depolymerization occurred within 30 min (**d**). At least three independent experiments were performed. Bar ± SEM

To investigate whether ProA could directly affect MT assembly and disassembly in a cell-free system, we tested the effect of ProA on tubulin polymerization in vitro by turbidimetry assay (OD, 350 nm)^18^. As shown in Fig. 4, ProA and digoxin did not display any significant effect on MT assembly whatever the concentration tested (Fig. 4a, b). In contrast, vinblastine, a MTA used as positive control, totally abolished polymerization at 20 µM (Fig. 4c). Furthermore, ProA when added to purified MTs under conditions favoring polymerization (37 °C and 1 mM GTP) failed to disassembly MTs (Fig. 4d). Thus, the disruption of the MT system observed in GBM cells did not result from an effect on MT polymer mass.

### NKA is required for the MT-dependent anticancer activity of Pro A

**Fig. 5:**
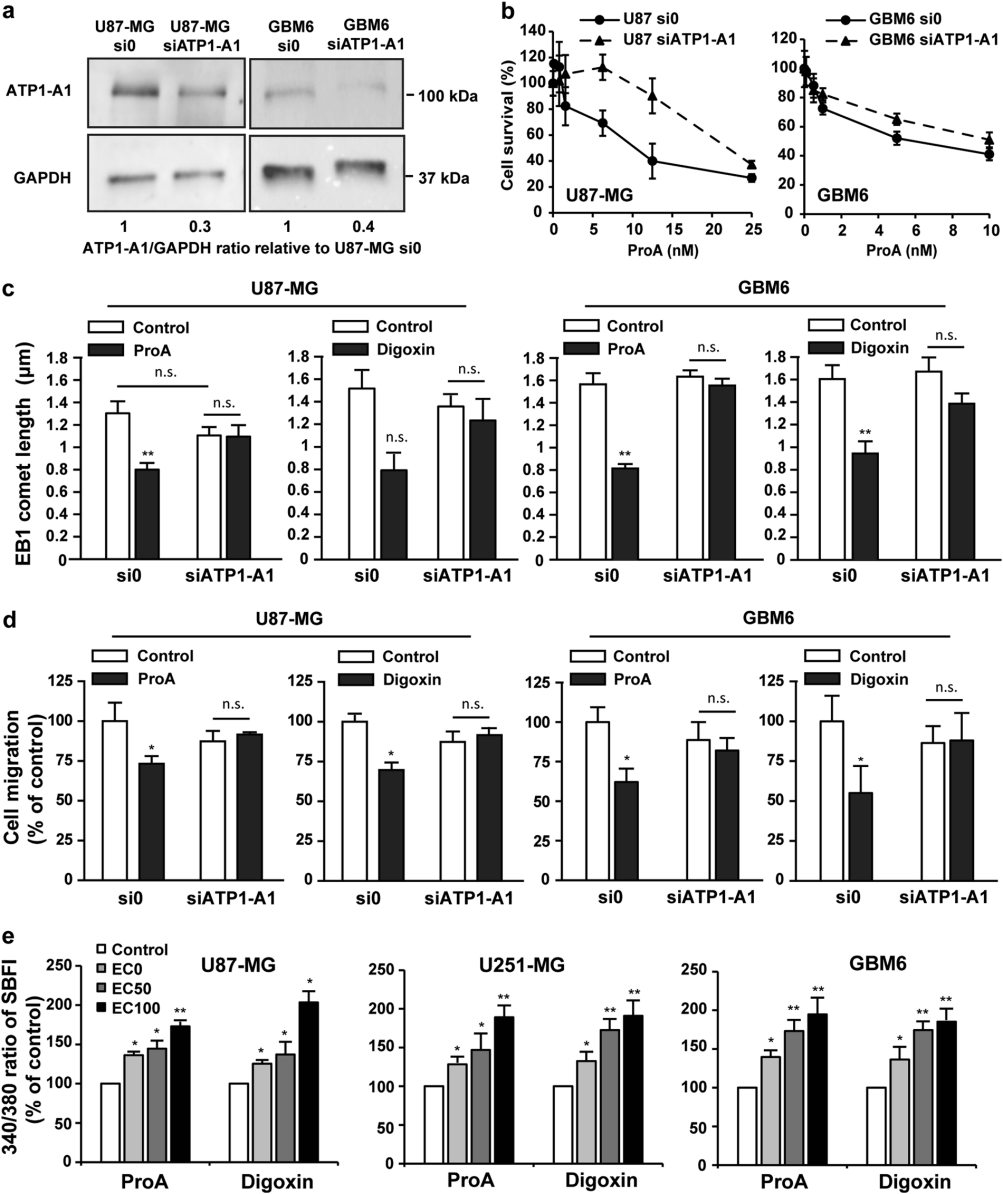
Cell migration inhibition and EB1 comet shortening induced by ProA were dependent on NKA-A1 activation. Western blot analysis of ATP1-A1 levels in U87-MG or GBM6 cells transfected with a siRNA against ATP1-A1 (siATP1-A1) or control empty vector (si0). Ratios ATP1A1/GAPDH, relative to control si0, from at least three independent experiments are presented under the blots (**a**). Dose-response curves of the cytotoxicity of ProA in U87-MG or GBM6 cells transfected with a siRNA against ATP1-A1 (siATP1-A1) or control empty vector (si0) (**b**). Quantification of EB1 comet length in U87-MG and GBM6 cells transfected with a siRNA against ATP1-A1 (siATP1-A1) or control empty vector (si0) and treated with vehicle (control), ProA or digoxin at EC50 for 5 h (**c**). Quantification of migratory U87-MG and GBM6 cells transfected with a siRNA against ATP1-A1 (siATP1-A1) or control empty vector (si0) and treated with vehicle (control), ProA or digoxin at EC50, for 5 h by transwell migration assay. Quantification was expressed as percentage of migrating cells relative to 100% of control cells (**d**). Percentage of intracellular sodium change by analysis of SBFI fluorescence (340/380 nm), in response to treatment with ProA, digoxin (at EC0, EC50, and EC100) or vehicle (control) in U87-MG, U251-MG, and GBM6 cells (**e**). At least three independent experiments were performed for each condition. Bar ± SEM. *p < 0.05; **p < 0.005; n.s., *p* > 0.05

Consequently, we asked whether NKA was needed to the indirect effect of CG on MTs by using siRNA against the α1 isoform of NKA (ATP1-A1) which is overexpressed in GBM cells as compared with normal brain tissue^19^. ATP1-A1 expression level (ratio to GAPDH) was strongly decreased in U87-MG-siATP1-A1 and in GBM6-siATP1-A1 cells (Fig. 5a) without consequence on EB1 comet length and cell migration, as compared with U87-MG si0 and GBM6 si0 (*p* > 0.05) (Fig. 5c, d, white bars) and U87-MG and GBM6 wild-type (not shown), respectively.

Interestingly, cytotoxic and antimigratory effects of ProA are decreased in U87-MG-siATP1-A1 cells and GBM6-siATP1-A1 cells as compared to the respective control si0 cells. Indeed, in U87-MG, EC50 were 6.24 ± 1.26 and 24.0 ± 1.51 nM for si0 and siATP1-A1 respectively and 3.28 ± 1.27 and 9.26 ± 1.16 nM for si0 and siATP1-A1 respectively, in GBM6 (Fig. 5b). Furthermore, both the decrease in EB1 comet length (Fig. 5c) and in cell migration (Fig. 5d) induced by ProA and digoxin at EC50 were abolished in U87-MG and in GBM6 under expressing ATP1-A1 cells. These results allow us to conclude that the MT effects of CG required ATP1-A1.

In cardiac myocytes, CG cause inhibition of NKA ATP1-A1 pump, leading to increased intracellular Na^+^ and consequently an impairment of sodium-dependent calcium transport out of cells^1^. Thus, we analyzed changes in 340/380 nm ratio of sodium indicator SBFI fluorescence after drug treatment at EC0, EC50, and EC100 in U87-MG, U251-MG, and GBM6 cells. As shown in Fig. 5e, ProA and digoxin activated Na+ influx in a dose-dependent manner, demonstrating that NKA was functional in GBM cells.

### EB1 phosphorylation and GSK3β activation mediated by ProA binding to NKA govern the reduction of EB1 comet length and the inhibition of cell migration

**Fig. 6:**
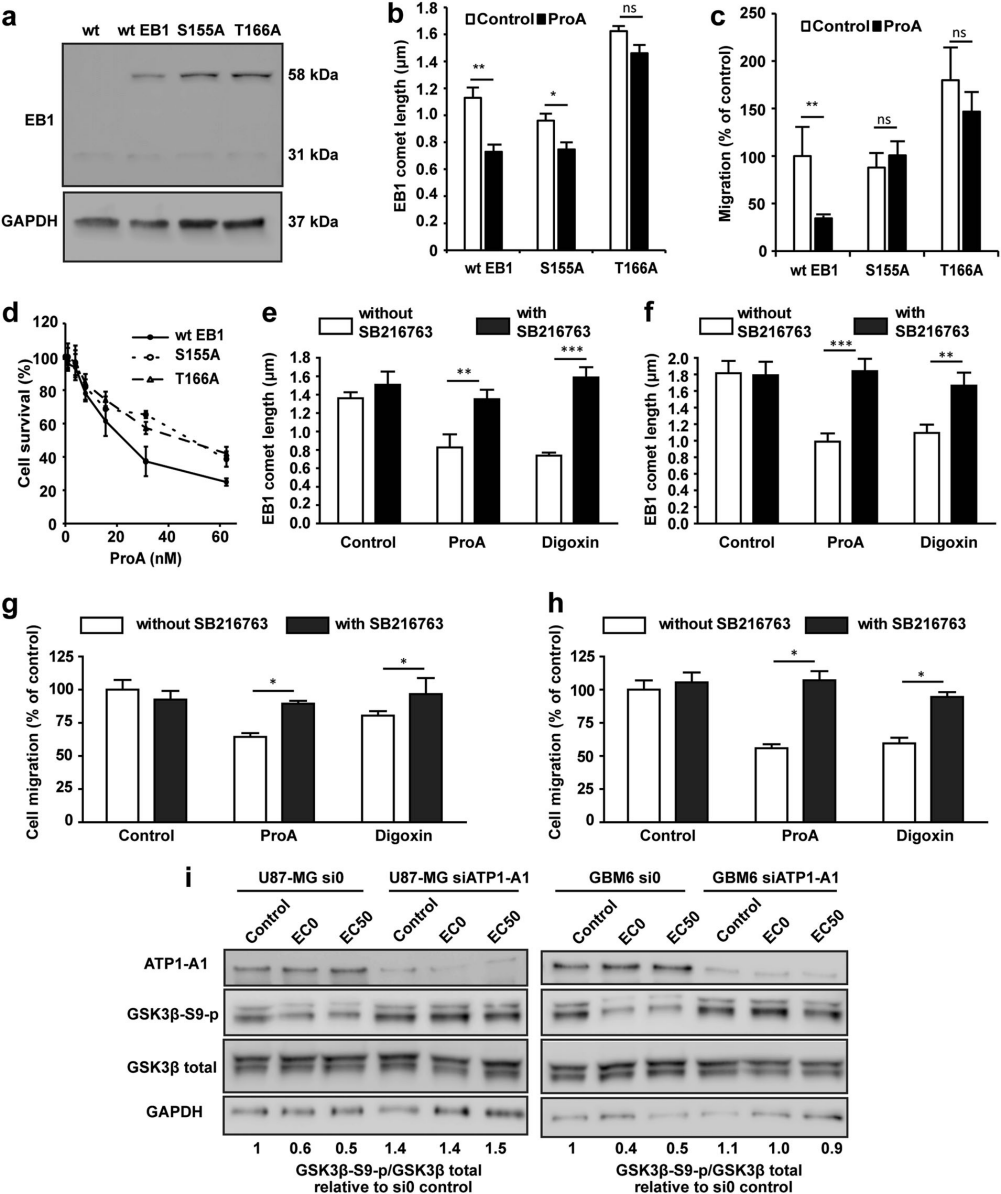
GSK3β was involved in the cell migration inhibition and EB1 comet shortening induced by ProA. Western blot analysis of EB1 expression in wtEB1-GFP, EB1 S155A-GFP, and EB1 T166A-GFP transfected U87-MG cells. U87-MG wild type (wt) was used as a negative control. EB1 expression analysis showed two protein bands for endogenous (31 kDa) and exogenous EB1-GFP (58 kDa) in the stably transfected cells. GAPDH was used as loading control (**a**). Quantification of EB1 comet length in wtEB1-GFP, EB1 S155A-GFP, and EB1 T166A-GFP transfected U87-MG cells treated with vehicle (control) or ProA at EC50 for 5 h (**b**). Quantification of migratory wtEB1-GFP, EB1 S155A-GFP, and EB1 T166A-GFP transfected U87-MG cells treated with vehicle (control) or ProA at EC50 for 5 h (**c**). Survival of wtEB1-GFP, EB1 S155A-GFP and EB1 T166A-GFP transfected U87-MG cells exposed to ProA, measured by the MTT test (**d**). Quantification of EB1 comet length in U87-MG (**e**) and GBM6 cells (**f**) treated or not with SB216763 and concomitantly exposed or not (control) to ProA or digoxin at EC50 for 5 h. Quantification of migratory U87-MG (**g**) and GBM6 cells (**h**) treated or not with SB216763 and concomitantly exposed or not (control) to ProA or digoxin at EC50 for 5 h, by transwell migration assay. Quantification was expressed as percentage of migrating cells relative to 100% of control cells. Western blot analysis of expression and activity of GSK3β under 5 h-treatment with ProA at EC0 or EC50 in U87-MG or GBM6 cells transfected with a siRNA against ATP1-A1 (siATP1-A1) or control empty vector (si0). Quantification of western blot bands was expressed as phospho/total GSK3β ratio. GAPDH was used as loading control (**i**). At least three independent experiments were performed for each condition. Bar ± SEM. *p < 0.05; **p < 0.005; ***p < 0.001; n.s., *p* > 0.05

Consequently, we hypothesize that low concentrations of CG activate a signaling pathway that could be NKA-dependent, leading to disruption of MT functions. EB proteins are regulated by post-translational modifications. Indeed, phosphorylation on serine 155 and threonine 166 of EB1 induced by GSK3β activation has been described to modulate MT dynamics, EB1 comet length, and cell migration^20^. To investigate whether this pathway is activated under CG treatment in GBM cells, we used U87-MG expressing phospho-defective human EB1 proteins, containing substitution of serine 155 or threonine 166 residues by an alanine residue. We first verified the overexpression of these phospho-defective EB1 proteins (EB1 S155A-GFP and EB1 T166A-GFP) or non-mutated EB1 protein (wtEB1-GFP control cells) in U87-MG cells (Fig. 6a). The decrease in EB1 comet length by ProA was totally inhibited in T166A mutant, and partially in S155A (Fig. 6b). Whatever the mutant, the strong antimigratory effect of ProA at EC50 was abolished (Fig. 6c). Moreover, as shown on Fig. 6d, ProA was statistically less cytotoxic on S155A and T166A cells, with EC50 values 1.5 and 1.7 fold-increased as compared to wtEB1 control cells (EC50 was 19.2 ± 2.5 nM, 22.1 ± 2.0 nM and 12.9 ± 1.1 nM for S155A, T166A, and wtEB1 cells respectively). These data strongly suggest that EB1 phosphorylation is involved in ProA anticancer effects.

Then, we assessed whether GSK3β activation by ProA was involved in EB1 delocalization and subsequent cell migration inhibition. The GSK3β specific inhibitor SB216763 restored EB1 accumulation at MT plus-ends in treated cells (Fig. 6e, f). Consistently, SB216763 suppressed cell migration inhibition induced by ProA at EC50 (Fig. 6g, h). Same results were obtained on U87-MG and GBM6 cell lines, and with digoxin at equipotent concentrations. Furthermore, ProA activated GSK3β as shown by the decrease of about 50% of expression of phosphorylated serine 9 GSK3β in U87-MG and GBM6 cells at EC0 and EC50 (Fig. 6i). Importantly, the decrease of S9 phosphorylation of GSK3β by ProA was abolished in underexpressing NKA ATP1-A1 U87-MG and GBM6 cells as compared with untreated control si0 cells. These results indicate that NKA expression is required for GSK3β activation by ProA.

Altogether, our data show that ProA activates a signaling pathway downstream of NKA, which is characterized by GSK3β activation, leading to EB1 phosphorylation, EB1 comet length shortening and MT dynamics alteration, and finally inhibition of cell migration and cytotoxicity.

## Discussion

Our work shows that low concentrations of ProA exert anticancer effects in GBM cells through inhibition of MT functions such as MT dynamic instability. Our study is the first showing a relationship between NKA activation, GSK3β activation, reduction of EB1 comet length at MT plus-end, and inhibition of GBM cell migration at sub-cytotoxic concentrations of ProA. Importantly, this mechanism of action is also demonstrated in cancer stem-like cells which are described to be at the origin of the tumor and to drive relapses due to their capacity of self-renewal and resistance to chemotherapy and radiotherapy^21–24^. Conversely, neural healthy cell lines such as oligodendrocytes and astrocytes were resistant to ProA. Drug specificity to cancer cells is essential for treating brain tumors, because maintaining a safe neurologic function is essential for quality of life.

Alteration of MT network by CG has been described in normal and cancer cell lines, mostly at high concentrations that induce G2/M arrest and apoptosis, but the underlying mechanism is poorly understood. Digitoxin showed a severely disruption and disorganized structure of MTs, and cell arrest in G2/M at concentrations that inhibit growth of lung cancer cells^25^. On the contrary, bufalin-induced mitotic arrest has been attributed to the downregulation of Plk1 expression, without alteration of MT polymerization in HeLa cells^26^. Fridman et al.^27^ described a distorded MT cytoskeleton with clusters of glycogen after treatment of human neuronal progenitor NT2 cells with bufalin, which results from a relocation of MTs and glycogen in the cell. Our findings establish CG as MT-diruptor agents at nanomolar concentrations in GBM as well as in other solid tumor cell types such as lung, breast cancer and neuroblastoma cells (personal data). Moreover, bufadienolides could be more potent than cardenolides.

Our study focused on low concentrations of ProA, which is more realistic for clinical applications. We show that ProA altered MT functions due to EB1 phosphorylation, subsequent inhibition of EB1 accumulation at MT plus-ends and alteration of MT dynamics. The reduction of EB1 comet length indicates a reduction of the EB1-stained MT-protective + end cap, which results in an increase in MT catastrophes and alteration of MT dynamics^28,29^. Interestingly, ProA modified MT distance-based catastrophe frequency, which is the main MT dynamic instability parameter that correlates with the inhibition of MT targeting to the cell cortex and consequently the inhibition of cell migration^17^. EB1 binding to MT and comet length are regulated through post-translational modifications of EB proteins such as phosphorylation^15,30^. Serine155 and Threonine166 are located in the EB1 linker region, which tightly contributes to its MT binding by promoting the Calponin-Homology domain binding^31,32^. Phosphorylation on the S155 residue is required for EB1 accumulation at MT plus-ends, and cancer cell migration. Phosphorylation of EB1 on T166 reduced comet length by decreasing MT growth rate and increased cancer cell migration. Here, we show that the shortening of EB1 comet and inhibition of cell migration by ProA were abolished in cells transfected with the phospho-defective S155A and T166A mutants, indicating that phosphorylation of S155 and T166 are requisite for antimigratory effect of ProA in cancer cells. EB1 phosphorylation may result from the serine/threonine kinase GSK3β phosphorylation, leading to its activation, which is required for the antimigratory effect of ProA and digoxin. GSK3 β activation by CG is downstream of NKA since it is no more observed in cells underexpressing NKA-A1 pump. GSK3β activation by bufalin and other CG such as ouabain and resibufogenin has been recently shown in liver, lung, and colon cell lines^33–36^.

The ion pumping function of NKA in the anticancer activity of CG is still under debate. Carr et al.^37^, have recently shown that EB1 comet shortening induced by electric fields was independent of calcium influx in U87-MG GBM cells. Conversely, elevation of transient spikes of calcium could activate calmodulin kinases, which in turn are known to affect GSK3β activity. At antimigratory concentrations, the potent anticancer MTAs also regulate MT dynamics by promoting GSK3β-mediated phosphorylation of EB1 in several cell lines, including GBM cells. However, only phosphorylation of T166 is required for MTA activity^30^. Moreover, MT dynamics alteration by ProA did not originate from an alteration of MT polymer mass contrary to classical MTAs. Some data suggest a potential link between NKA and tubulin. Indeed, tubulin in acetylated form interacts with plasma membrane NKA, resulting in inhibition of the enzyme activity, and detyrosinated tubulin also play a role in regulation of the enzyme, suggesting that compounds that bind to tubulin either in dimer or polymeric form could alter NKA activity^38,39^. Interestingly, paclitaxel, a well-known MTA, can inhibit in vitro the activity of the purified NKA in the same range of concentrations as digitoxin. Moreover, paclitaxel and digitoxin synergize to inhibit proliferation of Her2 overexpressing breast cancer cells. However, cumulative cardiac side effects should be considered for this drug combination^40^.

In GBM, one of the most common found abnormalities is the overexpression or aberrant activation of EGFR or its constitutively active mutant EGFRvIII^41^. In these tumors, the MAPK/Erk 1/2 and PI3K/Akt pathways have been identified as the driving forces of cellular proliferation and tumor progression. Activation of GSK3β by ProA may in part counteracts EGFR signaling, and contribute to the anticancer effect in GBM. Digoxin and digitoxin that belong to the group of CG called cardenolides has shown similar effects to ProA and bufadienolide that belong to the bufadienolide group, indicating that disturbance of MTs may be a common biological effect of this class of drugs. Advantages of toxicity and pharmacokinetic profiles may guide further clinical investigation.

Taken together, our results sustain the anticancer effects of ProA when administered by intra peritoneal route in mice orthotopically grafted with GBM and cancer stem-like cells^8^. CG at low concentrations, mimic the antimigratory and cytotoxic effects of MTAs although they bind to NKA and not to tubulin. However, the use of MTAs, which are very successful anti-cancer drugs in many solid tumors^42^, is restricted for GBM treatment due to the blood–brain barrier that blocks the crossing of most of the clinically relevant MTAs into the brain^43^. In conclusion, our studies support CG as an alternative treatment strategy and potent candidates for drug repositioning in GBM.

## Materials and methods

### Drugs

ProA (Sigma-Aldrich, Saint-Quentin Fallavier, France), digoxin (Sigma-Aldrich), bufalin (Merck, Fontenay-Sous-Bois, France) and digitoxin (Sigma-Aldrich) were dissolved at 18.8, 12.8, 10 and 10 mM respectively, in cell culture-grade dimethyl sulfoxide (DMSO) and stored at −20 °C until use. Vinblastine (Sigma-Aldrich) was solubilized in sterile distilled water. SB216763 (Sigma-Aldrich) was solubilized in DMSO. All these solutions were freshly diluted in the culture medium for experiments.

### Cell culture and transfection

Human GBM U87-MG, U251-MG cell lines and C8D1A mice astrocytes were ordered from ATCC. Cells were grown in DMEM media with glucose and L-glutamine (Lonza, Levallois-Perret, France), containing 10% fetal calf serum (Lonza), 1% penicillin/streptomycin (Sigma-Aldrich). Normal human astrocytes were ordered from Lonza and were cultured in AGM BulletKit astrocyte growth medium (Lonza). Oligodendroglial cell line OLN-93 was obtained from the Richter-Landsberg laboratory. These proliferating cells were routinely maintained as previously described^44^. Briefly, they were grown in DMEM-High Glucose-GlutaMAX (Lonza) supplemented by pyruvate (Lonza), 1% penicillin and streptomycin, 10% fetal bovine serum. GBM stem-like cell lines GBM6 and GBM9 were isolated from GBM patients and grown as previously described^9^. All cell types were tested weekly for the presence of mycoplasma, using Mycoalert^TM^ Mycoplasma Detection Kit (Lonza). Stable U87-MG clones overexpressing phospho-defective EB1 proteins (EB1-S155A-GFP and EB1-T166A-GFP U87-MG cells) or non-mutated EB1 proteins (wtEB1-GFP U87-MG cells) were generated as previously described^30^. For ATP1-A1 silencing by transient transfection, cells were transfected by lipofectamine 2000 system with siRNA for ATP1-A1 (Silencer Select siRNAs s1720, Thermo Fisher Scientific, NY, USA and Silencer Select negative control, Thermo Fisher Scientific). ATP1-A1 down-regulation was analyzed 72 h later by western blotting.

For analysis of MT dynamics, U87-MG cells (5 × 10^3^ cells/well) were grown for 24 h on 4-well Lab-tek Chambered Coverglass (Thermo Fisher Scientific). U87-MG cells were then transfected with plasmid coding for green fluorescent protein EB3-GFP using lipofectamine^TM^ 2000 system (Invitrogen, Villebon-sur-Yvette, France)^16^.

### Indirect immunofluorescence analysis

Cells were grown on 8-well chamber slides (Labtek, Thermo Fisher Scientific), precoated for 1 h with fibronectin (10 μg/ml) for U87-MG or with poly-DL-ornithine (Sigma-Aldrich) (10 µg/ml) for GBM6, to be treated for 6 h with ProA, digoxin, bufalin or digitoxin. As previously described^16^, cells were incubated with the anti-EB1 (clone 5; BD Biosciences, San Jose, CA) and α-tubulin (clone DM1A; Sigma-Aldrich) primary antibodies, and then with Alexa488 or 568-conjugated secondary antibodies (Invitrogen). Staining was observed using either a Leica DM-IRBE microscope or a Leica TCS SP5 confocal laser-scanning microscope (Leica, Heidelberg, Germany). Images were acquired using Metamoph software or the Leica Confocal software, and were processed using Image J software. For each experimental condition, at least 100 EB1 comets (in at least 10 cells) were examined to measure their length.

### Cytotoxicity assay

For 2D experiments, cells (5000 cells/well) were directly seeded in 96-well plates and allowed to grow for 24 h before treatment with ProA, digoxin, bufalin or digitoxin. GBM6 and GBM9 were seeded on poly-DL-ornithine (Sigma-Aldrich) coated 96-well plates (10 µg/mL). Growth inhibition of cells was measured after 72 h of treatment by using the colorimetric MTT assay (Sigma-Aldrich). Cell survival was expressed as a percentage as compared to DMSO control cells as previously described^14^.

To obtain U87–MG and GBM6 3D cell culture in a form of spheroids, cells growing in regular 2D cultures were rinsed once in phosphate buffered saline, then detached using 0.02% Trypsin/EDTA (Gibco, UK), pelleted by centrifugation at 200×*g* for 5 min and finally resuspended in a fresh culture medium supplemented with 20% methylcellulose and seeded on round-bottom 96-well plates (Greiner Bio-one, Courtaboeuf, France) at 1000 cells per well in 100 µL of medium as described previously^45^. After 72 h of growth at 37 ^◦^C under 5% CO_2_, spheroids were formed and 100 µL of fresh medium containing different amount of drugs with final concentrations from 0.4 to 10,000 nM were added to wells. Spheroid growth was maintained for 7 days by adding 10 µL of fresh medium per well three times a week and was monitored daily by measuring spheroid area on bright-field photomicrographs (Eclipse Ts2-FL, Nikon). All images were segmented using a custom macro script, written for ImageJ software. At day 7 of drug exposure, cell viability was assessed by resazurin reduction: 20 µl of resazurin reagent (alamarBlue, Thermo Fisher Scientific) were added to each well, and incubated for 18 h before fluorescence was read on a microplate reader (POLARstar Omega, BMG LABTECH) according to the manufacturer’s instructions. After subtraction of the fluorescence signal given by blank wells containing no cells, viability was calculated as a percentage of fluorescence observed in control wells. These experiments were repeated three times independently, with four wells per condition.

### Protein extraction and western blot analysis

Cells were lysed after 6 h treatment in RIPA buffer (Tris-HCl 50 mM pH 8.0, NaCl 250 mM, Triton-X100 0.1%) with a cocktail of proteases and phosphatases inhibitors (Sigma-Aldrich) added freshly. Protein concentrations were determined using the Bio-Rad Protein Assay (Bio-Rad laboratories, Marnes-la-Coquette, France). Thirty micrograms of total proteins were loaded into a 12% SDS-PAGE gel and electrotransferred onto a nitrocellulose membrane. Primary antibodies used were directed against EB1 (clone 5; BD Biosciences, San Jose, CA), ATP1-A1 (Proteintech Europe, Manchester, UK), Ser 9 phospho-GSK3β (Cell Signaling, Boston, USA), total GSK3β (Thermo Fisher Scientific) and GAPDH (Sigma-Aldrich). Peroxydase-conjugated secondary antibodies (Jackson Immunoresearch, Baltimore, USA) and chemiluminescence detection kit (Millipore, Molsheim, France) was used for visualization of protein bands. Chemiluminescent signal was acquired on a G:BOX imaging system (Syngene, Cambridge, UK) and quantification was done with Image J software.

### Transwell migration assay

Cells (5 × 10^4^ cells/well) were poured on the upper side of transwell migration chamber (Becton Dickinson, Le Pont de Claix, France) and allowed migrating for 5 h as previously described^16^. Six fields per condition were imaged and transmigrated cells were counted. Results were expressed as percent of transmigrated cells compared with no treatment condition. At least three independent experiments were performed for each condition.

### Analysis of MT dynamics from EB3-GFP comet data

Before time-lapse microscopy analysis, U87-MG-EB3-GFP cells were incubated with EC_50_ concentration of ProA or digoxin for 5 h. Following drug incubation, cells were maintained at 37 °C and placed in routine culture medium supplemented with 25 mM HEPES to reduce medium pH acidity during time-lapse analysis.

Time-lapse microscopy and image acquisition for MT dynamics experiments were performed with a LEICA TCS SP5 confocal microscope equipped with a 63X objective lens. Forty-five images per cell were acquired at 2-s intervals using a digital camera (CCD camera Hamamatsu) driven by LEICA LAS AF software.

Analysis of MT dynamic instability was done by tracking plus-ends EB3-GFP comets of individual MTs using the u-track 2.2.1 package (multiple-particle tracking MATLAB software)^46–48^. The software package can be downloaded from http://www.utsouthwestern.edu/labs/danuser/software/#utrack_anc. Comet detection requires no user intervention, as the detection algorithm automatically estimates locally optimal thresholds. Tracking and inference of complete MT trajectories by u-track requires user-defined settings of several control parameters (*Movie information*: Pixel size = 160 nm; Numerical aperture = 1,4; Camera bit depth = 8-bit; Time interval = 2 s. *Comet detection*: Watershed parameters = 3 (minimum threshold) and 1 (threshold step size) standard deviations; Difference of Gaussions filter parameters = 1 (low-pass gaussian standard deviation) and 4 (high-pass) pixels. *Tracking*: Maximum gap to close = 15 frames; Minimum length of track segments from first step = 4 frames; Maximum forward-backward angles = 30°−10°; Maximum shrinkage factor = 3; Fluctuation radius = 2 pixels; Tracking search radius range = 2–10 pixels. *Track analysis*: Forward gaps = unimodal thresholding; Backward = unimodal thresholding with comet latency correction.)

Correct tracking was verified by visual inspection of several overlay movies. The parameter settings were kept identical for all movies. We chose 5 metrics for comparative analyzes: growth speed, growth length, growth time, distance-based catastrophe frequency, and time-based catastrophe frequency. Importantly, distance and time-based catastrophe frequencies were calculated as the mean of the inverse growth length and growth time of each track. For each experimental condition (Control, ProA and digoxin), 14 to 16 cells were analyzed (10,000–13,000 comet tracks per condition).

All tracks were included in analysis of MT dynamics. We chose to keep in growth metrics the phases of attenuated dynamics as the resolution of the microscope and the automated tracking process allowed it. These phases are characterized by decreased growth length/speed and increased distance-based catastrophe frequencies.

### Tubulin polymerization assay

Tubulin was extracted and purified from lamb brains by Weisenberg procedure consisting in ammonium sulfate fractionation and ion exchange chromatography^49,50^. MTs were assembled by mixing 20 µM tubulin solution, 1 mM guanosine 5′-triphosphate (GTP), 4 mM MgCl_2_, 1 mM Ethylene Glycol Tetra-acetic Acid (EGTA), 2% DMSO in 80 mM PIPES-KOH buffer pH 6.8 and by raising the temperature to 37 °C. Polymerization of tubulin, which produced an increase of the optical density at 350 nm, was monitored by turbidimetry at 1 min intervals using a spectrophotometer (Polarstar omega, BMG Labtech)^51^.

### Intracellular free sodium analysis

U87-MG and U251-MG were seeded at 3 × 10^5^ cells/well on 6-well plates. GBM6 were seeded at the same density on poly-DL-ornithine (Sigma-Aldrich) coated 6-well plates (10 µg/mL). One day later, cells were treated with ProA or digoxin for 24 h. Then, cells were loaded with 5 µM sodium sensitive dye SBFI (Interchim, Montluçon, France). The incubation continued for 1 h at 37 °C. Then, cells were trypsinized and washed in fresh culture medium. After centrifugation (1200 rpm), cell pellets were resuspended in PBS and used within the next 2–4 h. Fluorescence was recorded from 1 mL aliquots of magnetically stirred cellular suspension at 37 °C using a Shimadzu spectrofluorophotometer with excitation wavelengths of 340 and 380 nm and emission at 505 nm. Changes in [Na^+^] were monitored by using the 340/380 nm fluorescence ratio according to the method of Grynkiewicz^52^.

### Statistical analysis

Each experiment was performed at least in triplicate. Data are presented as mean ± SEM. Cell counting, cellular viability data were analyzed by Student’s *t* test. Dynamic instability data were analyzed with the non-parametric Mann–Whitney test. Reported *p*-values are two-sided, and *p* < 0.05 was considered statistically significant. Asterisks indicate significant level vs control *p < 0.05; **p < 0.005; ***p < 0.001. Statistical analyses were performed with GraphPad 5.0 statistical software.

## Electronic supplementary material

Supplementary figure 1

Supplementary figure 2

Supplementary figure legends
