# Supplementary figures and images for: Proscillaridin A exerts anti-tumor effects through GSK3β activation and alteration of microtubule dynamics in glioblastoma
Source: Cell Death Dis. 2018 Sep 24;9(10):984. doi: 10.1038/s41419-018-1018-7 (PMC6155148; doi:10.1038/s41419-018-1018-7)

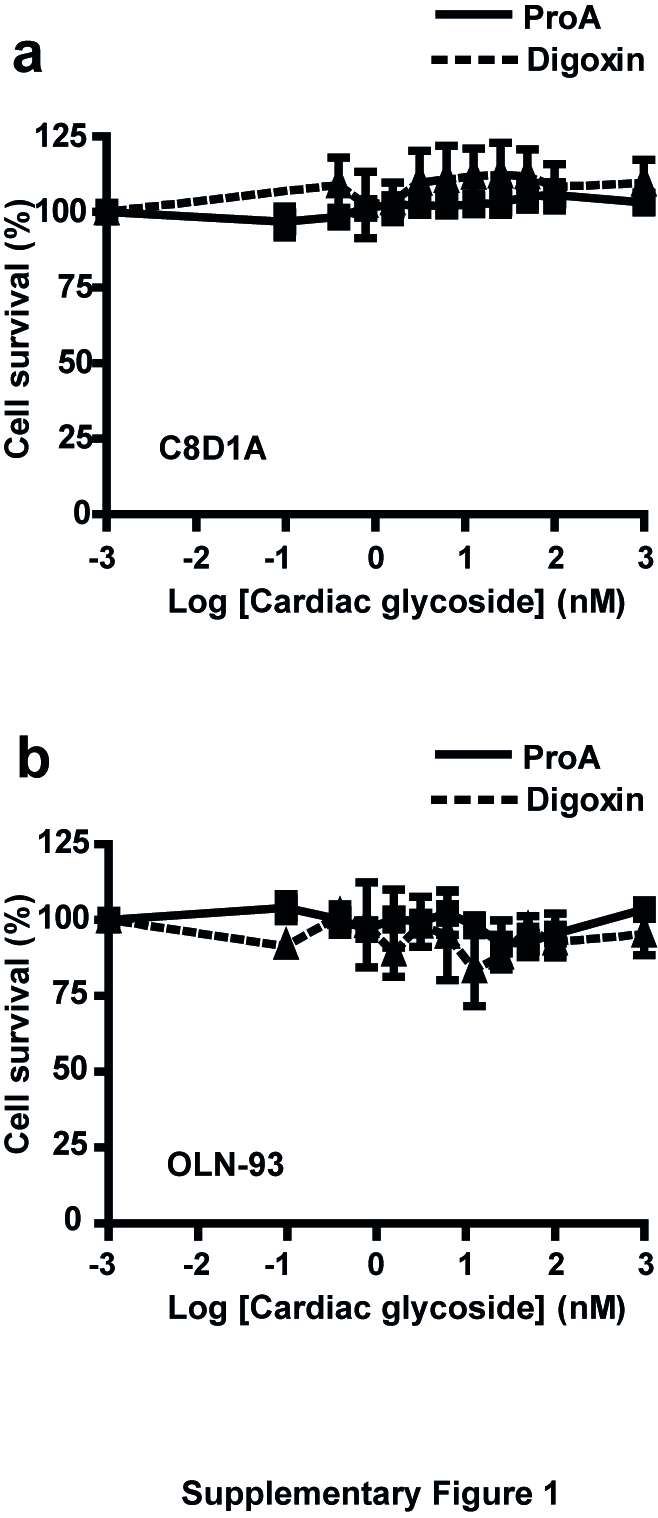

Supplement: Supplementary file 1 — Supplementary figure 1 [file 41419_2018_1018_MOESM1_ESM.tif]

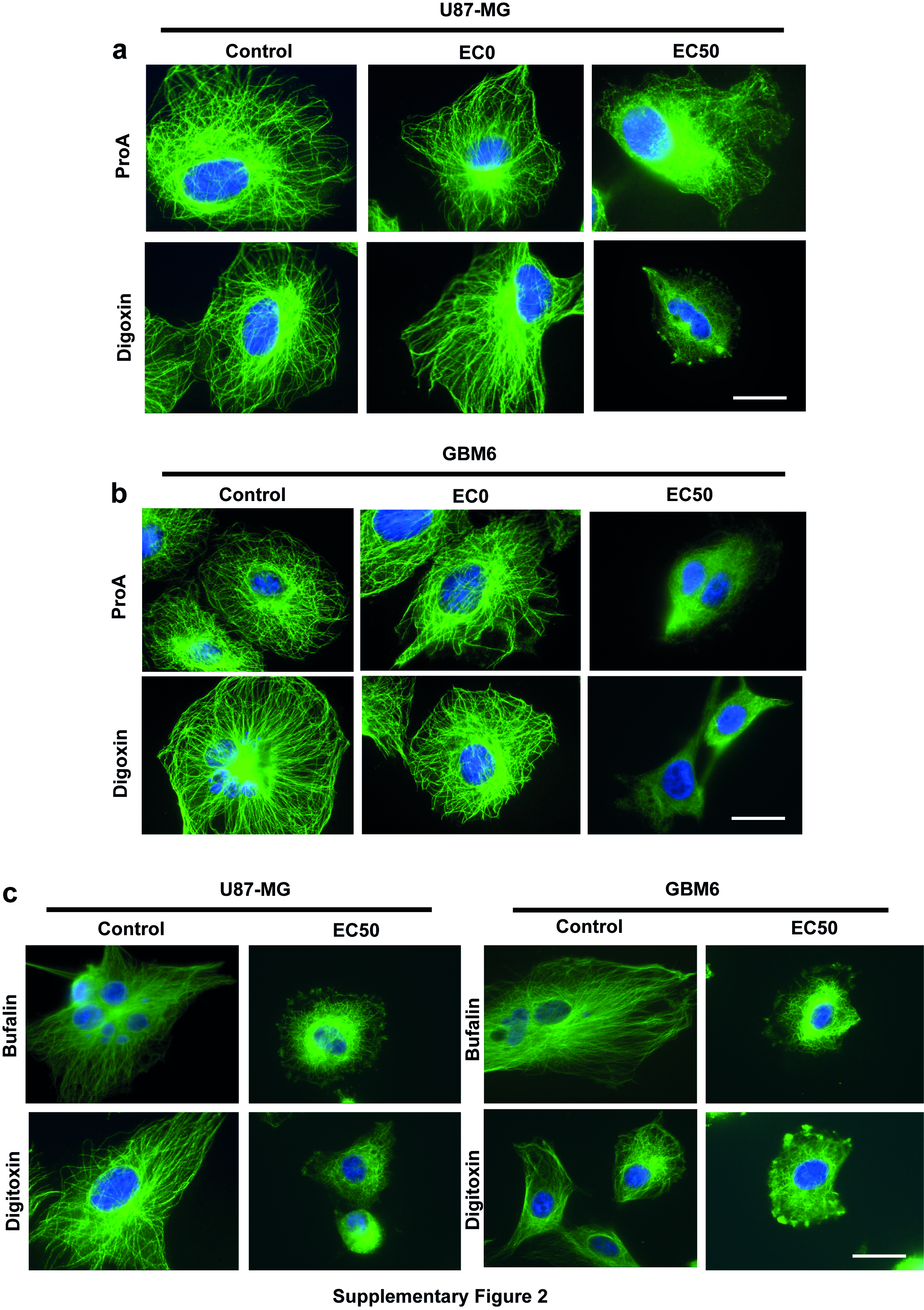

Supplement: Supplementary file 2 — Supplementary figure 2 [file 41419_2018_1018_MOESM2_ESM.tif]
