# Supplementary material for: Proscillaridin A exerts anti-tumor effects through GSK3β activation and alteration of microtubule dynamics in glioblastoma
Source: Cell Death Dis. 2018 Sep 24;9(10):984. doi: 10.1038/s41419-018-1018-7 (PMC6155148; doi:10.1038/s41419-018-1018-7)
Supplement: Supplementary file 3 — Supplementary figure legends [file 41419_2018_1018_MOESM3_ESM.docx]

**Supplementary Fig. 1: ProA did not display cytotoxic properties on healthy neural cells.** Dose response curves of the cytotoxicity of ProA and digoxin in C8D1A mouse astrocytes (**a**) and OLN-93 rat oligodendrocytes (**b**). At least three independent experiments were performed.

**Supplementary Fig. 2: Cardiac glycosides induced modifications of MT cytoskeleton architecture.** Immunofluorescence staining of α-tubulin in U87-MG (**a**) or GBM6 (**b**) treated or not (control) with ProA or digoxin at EC0 or EC50 for 5 h. Immunofluorescence staining of α-tubulin in U87-MG or GBM6 treated or not (control) with bufalin or digitoxin at EC50 for 5 h (**c**). At least three independent experiments were performed for each condition. *Bar=10 μm*.
